# Study on the impact of latent classes of ego depletion on self-management behaviors in older adults with hypertension

**DOI:** 10.3389/fpubh.2026.1771681

**Published:** 2026-03-12

**Authors:** Huixian Tang, Yongjun Chen, Bo Li, Ruixiang Sun, Liping Yuan

**Affiliations:** 1 Graduate School of Wannan Medical College, Wuhu, Anhui, China; 2 Department of Critical Care Medicine, Yijishan Hospital of Wannan Medical College, Wuhu, Anhui, China

**Keywords:** ego depletion, hypertension, influencing factors, latent class analysis, nursing, older adult, self-management behavior

## Abstract

**Objective:**

The study aimed to evaluate latent classes of ego depletion among older adults with hypertension and to analyze the association between different classes and self-management behaviors.

**Methods:**

A convenience sampling method was used to recruit 321 hospitalized older adults with hypertension from a tertiary hospital in Wuhu between May and November 2025. Data were collected using a general information questionnaire, the Self-Regulatory Fatigue Scale (to assess ego depletion), the Hypertension Self-Management Behavior Scale (to assess self-management level), the Medication Adherence Scale (to assess medication adherence), and the Chronic Disease Self-Efficacy Scale (to assess self-efficacy). The latent class analysis was performed to identify latent classes of ego depletion, while univariate and multiple logistic regression analyses were employed to explore the correlates of these classes and their association with self-management.

**Results:**

Ego depletion in older adults with hypertension can be divided into three latent classes, namely low depletion (38.63%), medium depletion (43.61%), and high depletion (17.76%). Primary caregivers, hypertension classification, and disease duration were key correlates of ego depletion, and self-efficacy was significantly associated with ego depletion classes (*p* < 0.05). However, there were significant differences in self-management levels among patients in different classes (*p* < 0.05).

**Conclusion:**

Older adults with hypertension exhibited relatively high levels of ego depletion with significant heterogeneity. Medical staff should recognize the heterogeneous characteristics and correlates of ego depletion and develop personalized interventions to alleviate ego depletion and improve patients’ self-management abilities.

## 1 Introduction

Primary hypertension is one of the most common chronic non-communicable diseases worldwide. The global prevalence among adults aged 30–79 years is 33% (approximately 1.3 billion people), according to age-standardized rates, (1). This condition has a particularly high incidence among older adults. The disease onset is associated with factors such as genetics, age, and unhealthy lifestyle habits. If left untreated, hypertension worsens over time and may lead to various cardiovascular and cerebrovascular diseases, such as atherosclerosis, stroke, and myocardial infarction, posing a serious threat to patients’ health. Research has shown (2) that patients with chronic diseases often experience significant ego depletion during long-term disease management. This phenomenon occurs when individuals expend excessive psychological resources in the ongoing self-control behaviors required to manage their illness, without adequate recovery time. As a result, they can reach a state of psychological resource exhaustion known as “ego depletion” (3). Ego depletion can result in negative outcomes, including reduced self-control, intensified negative emotions, and the resurgence of unhealthy habits (4). These consequences accelerate disease progression and reduce the overall quality of life. This state may lead to decreased self-regulation, accumulation of negative emotions, recurrence of adverse behaviors, and subsequent deterioration in health behavior, affecting treatment adherence, accelerating disease progression, and seriously impairing patients’ quality of life and long-term prognosis. Older adults with hypertension, due to cognitive decline, multiple comorbidities, reduced social support, and insufficient disease awareness, face a higher psychological burden in long-term self-management tasks, such as medication adherence, blood pressure monitoring, diet control, and exercise, making them more prone to ego depletion. However, present domestic research on self-loss in chronic disease patients largely employs traditional variable-centered analysis methods, focusing on the average effects across the population while neglecting individual differences and underlying heterogeneity in psychological resource consumption patterns. This limitation hinders the development of effective treatment strategies (5). Therefore, based on the above research background, this study breaks through the traditional assumption of homogeneity and shifts to an individual-centered perspective. The latent class analysis used in this study systematically identifies the latent class structure of ego depletion in older adults with hypertension, revealing distinct subgroup patterns in psychological exhaustion levels, manifestation characteristics, and combinations of risk factors. Furthermore, by constructing a latent class model and a longitudinal analysis framework, this study aims to deeply explore the impact of pathways and mechanisms of different depletion categories on patients’ self-management behaviors, such as medication adherence, lifestyle adjustments, and emotional regulation, providing a theoretical basis and practical reference for precisely improving patients’ self-management abilities. The ego depletion expression is consistent with the contemporary research literature in the field.

## 2 Objects and methods

### 2.1 Survey participants

Using convenience sampling, older adults with hypertension hospitalized in a tertiary general hospital in Wuhu from May to November 2025 were selected as the survey subjects. The inclusion criteria are as follows: ① Meet the hypertension diagnostic criteria according to the (6) European Society of Hypertension Guidelines (6), diastolic blood pressure≥90 mmHg or systolic blood pressure ≥140 mmHg; ② Age 60 years or older; ③ Clear consciousness and ability to communicate smoothly; and ④ Signed informed consent. The exclusion criteria are as follows: ① Severe mental illness preventing communication; ② In a coma, unable to provide any form of information or cooperate; and ③ Unwillingness to participate in this study. According to the sample size requirements for cross-sectional studies, the sample size should be 5–10 times the number of variables. This study included 28 independent variables, and considering a 10% invalid response rate, 156–311 cases should be included. Ultimately, 350 cases were surveyed. This study was approved by the hospital ethics committee, and all participants provided informed consent and voluntarily participated (Ethical Approval Number: (2025) Ethical Review for Research No. 321).

### 2.2 Survey tool

#### 2.2.1 General information survey form

The indicators for the general information survey form were set based on evidence-based medicine, referring to mainstream domestic and international literature and clinical research standards related to older adult hypertension. They systematically include sociodemographic variables closely related to the research topic and outcome measures, including age, gender, place of residence, primary caregiver, marital status, education level, personal monthly income, occupational status, living arrangements, smoking history, alcohol consumption history, exercise frequency, hypertension grade, duration of hypertension, use of antihypertensive medication, and presence of comorbidities.

#### 2.2.2 Self-regulatory fatigue scale

The scale was developed by Wang et al. (7), comprising three dimensions: cognitive control, emotional control, and behavioral control, with 16 items. The scale employs a 5-point Likert scale, with a total score ranging from 16 to 80 points. Items 3, 4, 6, 7, 8, 9, 11, 12, 13, 14, and 16 were positively scored (11 items in total), while Items 1, 2, 5, 10, and 15 were reverse-scored (5 items in total). For the reverse-scored items, score transformation was required: a raw score of 1 was converted to 5, 2 to 4, 4 to 2, and 5 to 1, with the raw score of 3 remaining unchanged. Higher scores indicate more severe ego depletion in patients. In this study, the scale demonstrated good measurement validity for older adults with hypertension. The confirmatory factor analysis supported the three-order factor model with a favorable fit (the root mean square error of approximation, RMSEA = 0.076 and the comparative fit index, CFI = 0.93), confirming its suitability for assessing self-regulatory fatigue in this population. The Cronbach’s *α* coefficient of this scale in this study was 0.921. Domestic and international relevant studies (8) had adopted these three dimensions as core indicators, forming an academic consensus and thus ensuring the comparability and generalizability of the findings of this study.

#### 2.2.3 Self-management behavior assessment scale for hypertensive patients

Developed by Zhao et al. (9), this 33-item scale comprises six dimensions: medication management, disease monitoring, dietary management, exercise management, work–rest balance, and emotional regulation. Using a 5-point Likert scale, the total score ranges from 33 to 165 points, with<60 indicating low self-management level, 60–80 as medium, and >80 as high. Higher scores reflect better self-management ability. The Cronbach’s *α* coefficient of this scale in this study was 0.858. The original study confirmed the scale had reliable validity: the overall content validity index (CVI) was 0.91, and the confirmatory factor analysis showed favorable model fit (RMSEA = 0.042, CFI = 0.956), meeting psychometric standards for both content and construct validity.

#### 2.2.4 Self-efficacy scale for chronic diseases

This scale was developed by Lorig et al. (10),comprising two dimensions: self-efficacy in symptom management and self-efficacy in disease management, with a total of six items. Each item is scored on a 1–10 scale from “completely unconfident” to “completely confident.” The total score is the average of all item scores, ranging from 1 to 10, where higher scores indicate greater patient self-efficacy. In this study, the validity of the chronic disease self-efficacy scale was confirmed in accordance with the original study. The CVI was 0.92. The confirmatory factor analysis demonstrated a well-fitting model (RMSEA = 0.058, CFI = 0.942) and therefore, the scale was deemed suitable for this study. The Cronbach’s *α* coefficient of this scale in this study was 0.821.

#### 2.2.5 General medication adherence scale

The general medication adherence scale (GMAS) was developed by Naqvi et al. (11). The scale includes 3 dimensions and 11 items, namely, patient behavior-related compliance (5 items), additional disease and medication burden (4 items), and cost-related compliance (2 items). Each item is scored 0, 1, 2, or 3 corresponding to “always,” “mostly,” “sometimes,” and “never,” respectively. The total score ranges from 0 to 33; a score of 30–33 indicates high patient compliance, 27–29 indicates good compliance, 17–26 indicates medium compliance, 11–16 indicates low compliance, and 0–10 indicates poor compliance. The Cronbach’s *α* coefficient of this scale in this study was 0.740. Validated in the original study, the GMAS for hypertensive patients demonstrated reliable validity: the overall CVI was 0.82, and confirmatory factor analysis showed a well-fitting model (RMSEA = 0.063, CFI = 0.92). Therefore, the scale was considered to be suitable for this study.

### 2.3 Data collection and quality control methods

Data collection and quality control were strictly implemented in accordance with standardized procedures. Based on systematic review of domestic and international research literature on hypertension in older adults, and combined with clinical practice, the influencing factors related to ego depletion fatigue in older adult hypertensive patients were identified. A specialized team composed of senior clinicians, specialist nurses, and researchers was formed. Postgraduate students who had undergone unified training conducted on-site data collection in a “one-on-one” manner, with only one patient’s information recorded per session. Throughout the process, guidance was provided to avoid arbitrary selection or omission, thereby ensuring the authenticity and completeness of the data. All collected forms were independently entered into Excel by two staff members within 6 h to create a parallel database. Built-in functions were used for field matching and logical validation, with any discrepancies identified during verification promptly traced and corrected. The database, verified by two individuals, was archived as the study data to ensure data accuracy. A total of 350 questionnaires were distributed in this survey, with 321 valid questionnaires recovered, yielding a valid response rate of 91.7%.

### 2.4 Statistical analysis

Using the three dimensions of the self-regulation fatigue scale (SRFS) for older adult hypertensive patients as exogenous variables, a latent class analysis (LCA) was conducted using Mplus 8.3 software. The data model was initially established from a single latent class category, with the number of categories gradually increased to identify the model with the best fit as the final model. The model fit indices were as follows: ① Akaike information criterion (AIC), Bayesian information criterion (BIC), and sample-corrected Bayesian information criterion (aBIC), where smaller values indicate better model fit; ② the Lo–Mendell–Rubin test (LMRT) and the Bootstrap likelihood ratio test (BLRT) were conducted to evaluate model fit differences, with *p* < 0.05 indicating that the k-category model was significantly superior to the k-1-category model; ③ Information entropy was employed to assess model classification accuracy, where values closer to 1 indicate higher precision, and entropy ≥ 0.8 indicated classification accuracy exceeding 90%. In this study, continuous scale scores were first converted into categorical variables, and then the LCA was performed to identify latent classes to meet the analytical requirements of categorical variables. The covariates in this study (e.g., age, gender, hypertension stage) were not included in the LCA model; instead, after identifying the latent classes, a multivariate logistic regression was conducted for post-hoc analysis to explore the associations between different latent classes and covariates. Statistical analysis was performed using SPSS25.0 software. Normally distributed quantitative data were expressed as means and standard deviations, with intergroup comparisons performed using the chi-squared test (χ^2^). Non-normally distributed quantitative data were expressed as medians and quartiles, with intergroup comparisons performed using the Kruskal–Wallis H test. Categorical data were expressed as counts, percentages, or percent rates, with intergroup comparisons performed using the χ^2^ test. Multivariate logistic regression analysis was conducted to investigate the influence of various factors on different latent classes, with a significance level of *α* = 0.05.

## 3 Results

### 3.1 Potential categories and nomenclature of Ego depletion in older adult hypertensive patients

**Figure 1:**
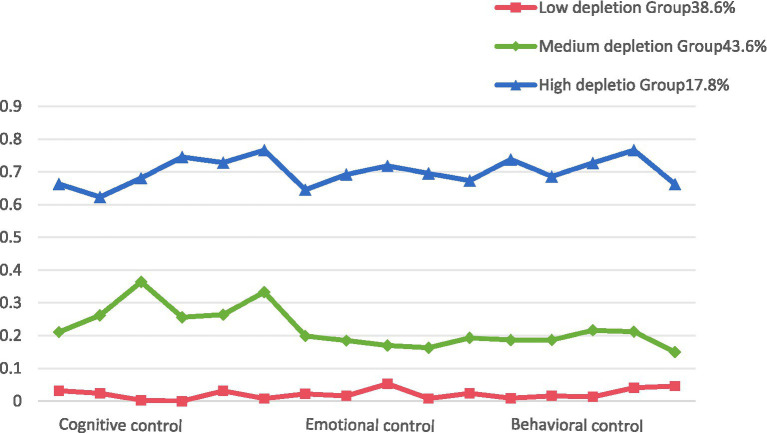
Characteristics distribution of three potential classes of ego depletion in older adults with hypertension. This figure shows the scores of cognitive control, emotional control, and behavioral control among the low, medium, and high ego depletion groups. Higher scores indicate better self-control ability.

**Figure 2:**
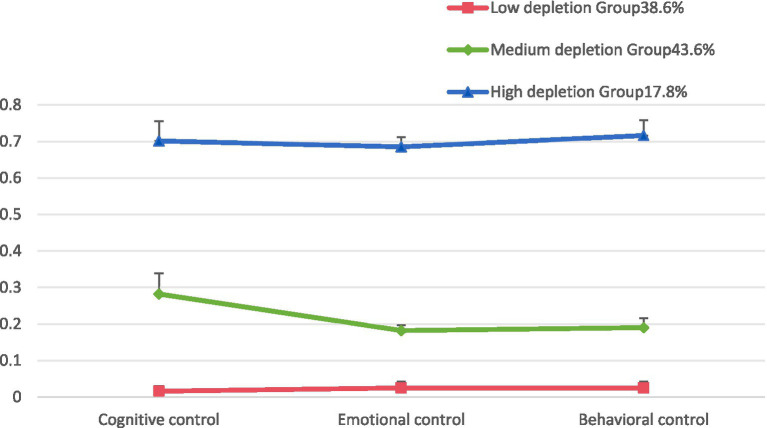
Average profile of cognitive, emotional, and behavioral control across three ego depletion groups (with error bars representing standard deviation). This figure illustrates the average scores of cognitive, emotional, and behavioral control among three ego depletion subgroups (low, medium, and high). Error bars represent the standard deviation (SD) of the scores, with higher scores indicating better self-control capacity. The participant distribution in each subgroup comprises low depletion group (38.6%), medium depletion group (43.6%), and high depletion group (17.8%).

The results of the latent class model for ego depletion in older adult patients with hypertension are presented in Table 1. AIC, BIC, and aBIC are core indicators for evaluating model fit, with smaller values indicating better model performance. The results showed that AIC, BIC, and aBIC all decreased with the increase in the number of latent classes. When divided into three classes, the entropy value reached its maximum, and the *p*-values of LMR and BLRT were <0.05. Although, the entropy value of the class 4 model was slightly higher than that of the class 3 model, further analysis revealed that the AIC, BIC, and aBIC values of the class 4 model increased compared to the class 3 model, and the *p*-values of the LMR and BLRT tests were greater than 0.05, indicating that adding a fourth category did not significantly improve model fit and was statistically insignificant. According to this study, a class 3 model was selected as the latent class analysis result for ego depletion in older adults with hypertension. Among them, class 3 (C3) exhibited generally higher scores across all items and was designated as the high depletion group, comprising 57 cases (17.76%). Class 1 (C1) showed lower scores across all items, indicating a milder degree of ego depletion, and was designated as the low depletion group, comprising 124 cases (38.63%). Class 2 (C2) had scores between the two groups and was designated as the medium depletion group, comprising 140 cases (43.61%) (see Figure 1). The scores of cognitive, emotional, and behavioral control differed significantly across the three ego depletion subgroups, with error bars indicating the variability within each group, and the detailed results are shown in Figure 2.

**Table 1:** Fitting metrics of the latent category model for ego depletion in older adults with hypertension (*n* = 321).

Class	AIC	BIC	aBIC	Entropy	*P*-value		Class proportion
					LMR	BLRT	
1	11037.009	11157.695	11056.196				1.00
2	9599.645	9844.789	9638.619	0.959	<0.001	<0.001	0.58/0.42
3	9182.239	9551.840	9241.000	0.966	<0.001	<0.001	0.44/0.18/0.38
4	9174.537	9668.596	9253.085	0.967	0.8981	0.8967	0.17/0.05/0.39/0.39

AIC stands for Akaike information criterion; BIC for Bayesian information criterion; aBIC for sample-corrected Bayesian information criterion; LMRT for the log-moments ratio test with Rouben correction; BLRT for the bootstrap-based likelihood ratio test. Values of class proportions refer to the proportion of each latent class in the total sample in the corresponding latent class model, reflecting the distribution of different latent classes in the study population.

### 3.2 Univariate analysis of ego depletion potential classes in older adults with hypertension

The results of this study showed statistically significant differences in age, education level, hypertension comorbidities, primary caregiver, hypertension classification, and disease duration among the three subgroups (*p* < 0.05), as shown in Table 2. The study subjects were predominantly aged 70–79 years (40.2%) and 60–69 years (33.3%). The education levels were mainly elementary school or below (40.5%) and junior high school (36.8%). Hypertension comorbidities were present in 95.3% of patients. The primary caregivers were children (39.8%) and spouses (34.3%). The most common hypertension classifications were stage 2 (34.3%) and stage 1 (30.2%). The disease duration was primarily 5–10 years (42.9%). The self-efficacy scores of patients in the low depletion, medium depletion, and high depletion groups were 32.68 ± 5.163, 31.59 ± 4.921, and 30.05 ± 4.712, respectively. The Levene’s homogeneity of variances test indicated homogeneity (*p* = 0.875), and the overall intergroup comparisons showed statistically significant differences (*F* = 5.246, *p* < 0.05).

**Table 2:** General characteristics and single-factor analysis of ego depletion potential classes in older adults with hypertension [cases (percentage,%)].

Project		Total number of cases (*n* = 321)	Low depletion group (*n* = 124)	Medium depletion group (*n* = 140)	High depletion group (*n* = 57)	χ2 value	*P* value
Age	60–69 years old	107 (33.3)	64 (51.6)	35 (25.0)	8 (14.0)	55.675	<0.001
	70–79 years old	129 (40.2)	41 (33.1)	71 (50.7)	17 (29.8)		
	80–89 years old	80 (24.9)	18 (14.5)	33 (23.6)	29 (50.9)		
	Over 90 years old	5 (1.6)	1 (0.8)	1 (0.7)	3 (5.3)		
Educational level	Primary school and below	130 (40.5)	24 (19.4)	72 (51.4)	34 (59.6)	41.078	<0.001
	Junior middle school	118 (36.8)	59 (47.6)	44 (31.4)	15 (26.3)		
	High school/college	60 (18.7)	35 (28.2)	20 (14.3)	5 (8.8)		
	Bachelor’s degree or above	13 (4.0)	6 (4.8)	4 (2.9)	3 (5.3)		
Comorbidities of hypertension	Have	306 (95.3)	118 (95.2)	132 (94.3)	56 (98.2)	263.804	<0.001
	Not have	15 (4.7)	6 (4.8)	8 (5.7)	1 (1.8)		
Primary caregiver	Spouse	110 (34.3)	76 (61.3)	28 (20.0)	6 (10.6)	79.611	<0.001
	Oneself	52 (16.2)	24 (19.4)	21 (15.0)	7 (12.3)		
	Father and mother	31 (9.7)	10 (8.1)	18 (12.9)	3 (5.3)		
	Sons and daughters	128 (39.8)	14 (11.2)	73 (52.1)	41 (71.8)		
Hypertension classification	Level 1	97 (30.2)	60 (48.4)	30 (21.4)	7 (12.3)	116.016	<0.001
	Level 2	110 (34.3)	52 (41.9)	54 (38.6)	4 (7.1)		
	Level 3	82 (25.5)	10 (8.1)	47 (33.6)	25 (43.9)		
	Above Level 3	32 (10.0)	2 (1.6)	9 (6.4)	21 (36.7)		
Hypertension course	Less than 1 year	59 (18.4)	44 (35.5)	14 (10.0)	1 (1.8)	142.030	<0.001
	1–5 years	77 (23.9)	50 (40.3)	24 (17.1)	3 (5.3)		
	5–10 years	138 (42.9)	28 (22.6)	85 (60.7)	25 (43.9)		
	More than 10 years	47 (14.8)	2 (1.6)	17 (12.2)	28 (49.0)		

### 3.3 Multifactorial analysis of ego depletion potential classes in older adults with hypertension

A multivariate logistic regression analysis was conducted with three potential categories (C1–C3) as dependent variables and statistically significant indicators from the univariate analysis as independent variables. The variable assignment is shown in Table 3, and the results of the multivariate logistic regression analysis are presented in Table 4. The results indicate that among the study subjects, hypertension was more prevalent in grade 2 (34.3%) and grade 1 (30.2%). The disease duration was primarily concentrated in the 5–10 year interval (42.9%). Age distribution was mainly 70–79 years (40.2%) and 60–69 years (33.3%). Educational levels were predominantly elementary school or below (40.5%) and junior high school (36.8%). Hypertension comorbidities were present in 95.3% of patients. Among primary caregivers, children (39.8%) and spouses (34.3%) ranked highest. This multivariate analysis focused on outcome correlates among groups C1, C2, and C3, involving four major categories of variables: chronic disease self-efficacy total score, primary caregivers, hypertension grade, and disease duration. Among these, when comparing C1 with C2, the total self-efficacy score for chronic diseases, the spouse as the primary caregiver, the classification of grade 1 hypertension, and the disease course status exert negative associations. When comparing C1 with C3, the primary caregiver being oneself or parents, the classification of grade 1–2 hypertension, and the disease course exert negative effects. In contrast, when comparing C2 with C3, only the classification of grade 3 hypertension and the disease course exert negative association with the outcome.

**Table 3:** Variable assignment method.

Variables	Assignment method
Age	60–69 years = 1, 70–79 years = 2, 80–89 years = 3, and ≥90 years = 4
Educational level	Primary school or below = 1, middle school = 2, high school/diploma = 3, and bachelor’s degree or above = 4
Comorbidities of hypertension	Yes = 1, No = 2
Primary caregiver	Spouse = (0, 0, 0), Self = (1, 0, 0), Parents = (0, 1, 0), Children = (0, 0, 1)
Hypertension classification	Level 1 = 1, Level 2 = 2, Level 3 = 3, Levels above 3 = 4
Hypertension course	<1 year = 1, 1–5 years = 2, 5–10 years = 3, over 10 years = 4
Self-efficacy score	Measured value
Self-efficacy potential classes category	Low depletion group = 1, medium depletion group = 2, and high depletion group = 3

**Table 4:** Multivariate logistic regression analysis of factors associated with ego depletion latent classes in older adults with hypertension.

Group	Project	β-Value	Standard error	Wald χ^2^ Value	*P* value	OR value	95%CI
Comparison of C1 and C2^1)^	Constant	−9.932	1.917	26.844	0	—	—
	Total self-efficacy score for chronic diseases	−0.066	0.027	5.879	<0.05	0.936	0.880–0.995
	Primary caregiver (spouse)	−2.237	0.379	34.824	0	0.107	0.049–0.235
	Hypertension classification (Grade 1)	−3.174	0.571	30.856	0	0.042	0.013–0.136
	Hypertension course (Grade 1)	−4.252	0.596	50.964	0	0.014	0.003–0.057
Comparison of C1 and C3^1)^	Constant	−9.932	1.917	26.844	0	—	—
	Primary caregiver (self)	−1.616	0.418	14.954	0	0.2	0.092–0.436
	Primary caregiver (parent)	−1.105	0.479	5.317	<0.05	0.331	0.119–0.926
	Hypertension classification (Grade 1)	−3.174	0.571	30.856	0	0.042	0.013–0.136
	Hypertension classification (Grade 2)	−2.554	0.548	21.693	0	0.078	0.024–0.254
	Hypertension course (Grade 1)	−4.252	0.596	50.964	0	0.014	0.003–0.057
	Hypertension course (Grade 2)	−3.526	0.546	41.678	0	0.029	0.008–0.104
Comparison between C2 and C3^2)^	Constant	−5.264	1.821	8.359	<0.05	—	—
	Hypertension classification (Grade 3)	−1.139	0.529	4.638	<0.05	0.32	0.108–0.940
	Hypertension course (Grade 3)	−1.855	0.455	16.652	0	0.156	0.064–0.381

C1 refers to the low depletion group; C2 refers to the medium depletion group; C3 refers to the high depletion group. Superscript 1) indicates the comparison with C1 as the reference group, and superscript 2) indicates the comparison with C2 as the reference group.

### 3.4 Comparison of self-management levels among older adults with hypertension in different ego depletion subgroups

Comparison results of the self-management scale scores among older adults with hypertension showed statistically significant overall intergroup differences in total self-management scores among the three groups (H = 40.942, *p* < 0.001). Intergroup difference analysis for each dimension demonstrated that statistically significant intergroup differences existed in medication management (H = 59.672, *p* < 0.001), disease monitoring management (H = 11.725, *p* < 0.05), dietary management (H = 25.622, *p* < 0.001), and emotional management (H = 11.825, *p* < 0.05), while no statistically significant intergroup differences were found in sports management (H = 47.729, *p* = 0.052) and rest management (H = 17.089, *p* = 0.065). The results of multiple comparisons concluded that the low ego depletion group and medium ego depletion group, when compared separately with the high ego depletion group, as well as the low ego depletion group compared with the moderate ego depletion group, exhibited statistically significant differences in medication management, disease monitoring management, dietary management, emotional management, physical activity management dimensions, and total self-management score (*p* < 0.05), with only the rest management dimension showing no statistically significant differences among groups (*p* > 0.05). In conclusion, the ego depletion level of older adults with hypertension was negatively correlated with their self-management level, that is, the higher the ego depletion level, the lower the self-management level; this negative correlation was stably reflected in the majority of self-management dimensions and the overall self-management level, whereas no obvious significant intergroup differences were observed in physical activity and rest management dimensions (see Table 5).

**Table 5:** Comparison of self-management scores among different subgroups of ego depletion in older adults with hypertension [points, M(P25, P75)].

Class	Number of cases	Medication management	Condition monitoring and management	Dietary management	Sports management	Rest management	Emotion management	Total self-management score
Low depletion group	140	20.00 (18.00, 22.00)^1)2)^	18.00 (16.00, 20.00)^1)2)^	17.00 (15.00, 19.00)^1)2)^	16.00 (14.00, 18.00)^1)2)^	20.00 (18.00, 22.00)	20.00 (18.00, 22.00)^1)2)^	120.00 (108.00, 132.00)^1)2)^
Medium depletion group	124	18.00 (16.00, 20.00)^1)^	17.00 (15.00, 19.00)^1)^	17.00 (15.00, 19.00)^1)^	16.00 (14.00, 18.00)^1)^	16.00 (14.00, 18.00)	17.00 (15.00, 19.00)^1)^	100.00 (88.00, 112.00)^1)^
High depletion group	57	14.00 (12.00, 16.00)	16.00 (14.00, 18.00)	14.00 (12.00, 16.00)	14.00 (12.00, 16.00)	16.00 (14.00, 18.00)	14.00 (12.00, 16.00)	88.00 (76.00, 100.00)
H price		59.672	11.725	25.622	47.729	17.089	11.825	40.942
P price		<0.001	0.003	<0.001	0.052	0.065	0.003	<0.001

1) Compared to the adaptive high depletion group, *p* < 0.05; 2) Compared to the symptom-disturbed medium depletion group, *p* < 0.05.

### 3.5 Intergroup comparison of medication adherence

The results of one-way ANOVA depicted no significant difference in medication adherence scores among the three groups (*F* = 0.345, *p* > 0.05). The mean scores of the three groups were 4.43, 4.54, and 4.58, respectively, with an overall mean of 4.50. The results suggest that despite the differences in ego depletion levels across groups, there was consistency in basic medication-taking behavior. However, significant intergroup differences were observed in the “medication management” dimension of the self-management scale (*p* < 0.05). Such differences stemmed from the essential distinction between the two concepts: medication adherence only measures the basic behavior of “taking medication on time and in the correct dose”, while the “medication management” dimension covers more complex active health management behaviors, including active blood pressure monitoring, communicating with medical staff about medication effects, and addressing side effects. The results conclude that ego depletion mainly affects patients’ active health management behaviors rather than basic medication-taking behaviors. Although medication adherence showed no statistical significance, the specific *p* value (*p* < 0.05) was reported to ensure transparency.

## 4 Discussion

### 4.1 Older adults with hypertension exhibit higher levels of ego depletion exhaustion with persistently severe trends

Hypertension is a key chronic disease affecting the health of the aging population. In China, the prevalence rate among individuals aged 60 years and above is 58.9%, yet the rate of achieving target control is only 32.2% (12). Ego depletion, as a state of exhaustion of self-regulatory resources, not only weakens patients ‘adherence to healthy behaviors such as dietary control and regular medication, increasing the risk of cardiovascular complications, but also forms a vicious cycle with negative emotions, exacerbating the burden of medical care and family caregiving pressure. These hazards exhibit multidimensional chain effects. In this study, the latent class analysis revealed that the total score of the ego depletion scale in older adults with hypertension was 43.09 ± 14.43, which was statistically significant (*p* < 0.05) and at a medium level, consistent with the findings of Tao et al. (13) using the self-regulatory fatigue scale. Given the prolonged course of hypertension and insufficient understanding of their own disease, older adults are highly susceptible to negative emotions such as anxiety and depression during treatment (14), thereby reducing treatment adherence and severely impacting disease prognosis. The latent class analysis results indicated that ego depletion in older adults with hypertension could be categorized into three groups: low depletion group (38.63%), medium depletion group (43.61%), and high depletion group (17.76%). Among these, the high depletion group accounted for the smallest proportion, while the low depletion and medium depletion groups accounted for the largest proportions, fully demonstrating the significant heterogeneity of ego depletion in hypertensive patients. Hypertension is a chronic and insidious disease (15), with the majority of the patients exhibiting subtle symptoms that often fail to attract sufficient attention during the early stages. The subjective pressure and behavioral constraints in daily management are relatively weak, and the consumption of self-control resources is minimal. Although the proportion of high depletion groups is low, ego depletion directly impacts the health behaviors of chronic disease patients. Therefore, healthcare professionals must emphasize stratified assessments: for medium depletion groups, disease awareness should be reinforced, and management processes simplified; for high depletion groups, individualized support plans should be developed, with health education to enhance their awareness and mitigate the negative associated with deterioration on self-management, thereby improving long-term prognosis. This study selected patients from a single tertiary Grade A hospital as survey subjects, resulting in a relatively limited scope of research.

### 4.2 Analysis of correlated on ego depletion potential classes categories in older adults with hypertension

#### 4.2.1 Caregiver factors

In the management of chronic diseases, caregiver type serves as a critical social support, influencing patients’ psychological state and self-depletion levels, with its correlates closely related to the disease stage and psychological resource regulation capacity of the patients (16). Data from this study indicate that older adults with hypertension with primary caregivers being their spouses account for 34.3%, and this group exhibits a significantly higher probability of belonging to the low depletion group. Moreover, during the disease onset, progression, or acute phases, the incidence of self-depletion among patients with spouses as primary caregivers (61.3%) in the low depletion group is higher compared to those with children as caregivers (11.2%) or parents as caregivers (8.1%). The underlying mechanism of this result may be that spouses, as long-term cohabiting caregivers, are more willing to actively share patients’ genuine feelings during the early or acute stages of the disease. Intimate communication between spouses not only provides patients with a sense of emotional belonging and targeted psychological support but also enhances their psychological resilience through emotional resonance in intimate relationships, alleviating negative emotions such as anxiety and depression (17). Consequently, patients’ intrinsic motivation for self-regulation is strengthened, reducing ego depletion. Therefore, in clinical practice, healthcare professionals should emphasize the regulatory role of caregiver types on patient ego depletion. For older adults with hypertension, priority should be given to encourage spouses to participate in disease care. Meanwhile, considering the family structure characteristics of patients, a diversified care model should be established with “spouses as primary caregivers and children/relatives as supplementary caregivers”. By enhancing the communication skills and psychological support capabilities of spouses, the positive role of spousal care in reducing patient ego depletion can be further leveraged.

In the psychological resource management of older adults with chronic diseases, effective expression and release of emotions are critical to avoiding potential depletion of psychological resources. However, relationship differences between caregivers and patients due to generational gaps often influence patients’ willingness to express emotions (18). In this study, older adults with hypertension whose primary caregivers are their children accounted for 39.8%, a group more likely to be categorized as middle or high depletion. The underlying reason is that patients may impose life and financial burdens on their children due to their own illness, leading to deliberately suppress negative emotions. To address this, clinical interventions should provide psychosocial resource support for patients cared for by children from multiple dimensions. On the one hand, for patients without spousal care and primarily supported by their children, healthcare providers should establish early ego depletion risk warning mechanisms through regular psychological assessments to identify early signs of ego depletion (19). On the other hand, expanding emotional expression channels for patients, such as organizing mutual aid groups for older adults with hypertension or providing one-on-one psychological counseling services, guiding patients to actively express their needs and inner feelings to healthcare providers, friends, or peer groups, thereby reducing the probability of early ego depletion.

#### 4.2.2 Disease duration factors in hypertension

In the chronic disease management of older adults with hypertension, disease duration serves as a key indicator reflecting disease progression and patient adaptation status, showing significant correlation with psychological resource depletion. Persistent physical discomfort resulting from long disease duration is an important contributing factor to psychological resource depletion and the decline in patients’ self-sustaining ability (20). Among the 321 patients enrolled in this study, 42.9% had a disease duration of 5–10 years, while 14.8% had a duration exceeding 10 years. In the high depletion group, the proportions of patients with 5–10 years and over 10 years of disease duration reached 43.9 and 49.0%, respectively, which are significantly higher than those in the low depletion group (22.6 and 1.6%, *p* < 0.05). The longer the course of hypertension, the higher the risk of high ego depletion in older adults, which is closely related to cumulative pathophysiological damage caused by long-term illness and continuous consumption of self-management resources (21). The prolonged course is accompanied by vascular remodeling, progression of target organ damage, and increased arterial stiffness. In addition, older adults often exhibit elevated pressure perception sensitivity, while long-term blood pressure fluctuations and physical discomfort continuously occupy psychological regulatory resources. Hypertension also requires lifelong adherence to medication compliance, a low-salt diet, regular monitoring, and other self-management behaviors. The longer the course of the disease, the more significant the burnout of patients towards long-term health constraints, and the rate of self-control resource consumption far exceeds that of supplementation, resulting in an imbalance state. Moreover, patients with a disease course exceeding 10 years are prone to mild cognitive decline, necessitating greater psychological resources to maintain complete disease management, which further exacerbates self-regulatory fatigue and ultimately leads to elevated ego depletion levels (22).

To address this phenomenon, clinical practice can alleviate ego depletion in older adults with a long course of hypertension through basic intervention measures. First, simplifying the disease self-management process and breaking down long-term health behaviors into simple and executable daily tasks can reduce the difficulty of implementation and the psychological burden, thereby decreasing the excessive resource consumption associated with complex health management strategies. It is important to prioritize psychological counseling for these patients, alleviate their anxiety about disease progression and complications through patient communication, and reduce the depletion of psychological resources induced by chronic psychological stress (23). In addition, patients should be encouraged to follow regular work and rest patterns and engage in moderate, gentle exercise in daily life. These habits may improve physical discomfort and emotional wellbeing, enhance psychological resilience, and strengthen their self-regulation capacity.

#### 4.2.3 Hypertension classification factors

Among the 321 participants included in this study, 30.2% had grade 1 hypertension, 34.3% had grade 2 hypertension, and 25.5% had grade 3 hypertension. Group analysis revealed that the high depletion group had the lowest proportion of patients with grade 2 hypertension (7.1%), which was statistically significant compared to the low depletion group (41.9%) (*p* < 0.05). Grade 1 hypertension accounted for 12.3% in the high depletion group that was lower than the low depletion group (48.4%), but the intergroup difference was not statistically significant (*p* > 0.05). Grade 3 hypertension accounted for only 8.1% in the low depletion group, also showing no statistically significant difference compared to the high depletion group (43.9%) (*p* > 0.05). The results of this study indicate that older adults with hypertension with higher grade classifications are more likely to belong to the high depletion group. Furthermore, these patients often experience more pronounced somatic symptoms, such as headache and chest tightness, and the frequent onset of symptoms rapidly depletes their psychological resources. Additionally, higher-grade hypertension imposes stricter requirements on diet, exercise, and medication, necessitating sustained self-discipline from patients, which further accelerates the depletion of psychological resources (24). Furthermore, patients with higher-grade hypertension exhibit greater concerns about disease complications, and this persistent negative emotion continuously consumes their self-regulation capacity, making it more difficult for them to maintain a good psychological state and gradually falling into a high depletion state (25). Therefore, for older adults with hypertension with higher grade classifications, psychological support and simplified management interventions should be implemented alongside blood pressure control to help alleviate ego depletion.

#### 4.2.4 Self-efficacy factors in chronic diseases

The self-efficacy scores of patients in the low depletion group, medium depletion group, and high depletion group were 32.68 ± 5.163, 31.59 ± 4.921, and 30.05 ± 4.712, respectively. The Levene’s test confirmed homogeneity of variances (*p* = 0.875), and the overall intergroup comparison was statistically significant (*F* = 5.246, *p* < 0.05). These results indicate that the total score of chronic disease self-efficacy is negatively correlated with ego depletion risk. Hypertensive patients with higher self-efficacy are more likely to attribute their outcomes to the low depletion group, which is consistent with the findings of Lin et al. (26). Older adults with hypertension who have high self-efficacy often demonstrate stronger psychological resilience and adaptive recovery capabilities when facing disease-related challenges, such as social role adjustments and changes in daily routines. These patients possess more robust intrinsic motivation for self-regulation and richer psychological resources, enabling them to effectively cope with various pressures and challenges in daily life caused by the disease. From the perspective of disease management characteristics, older adults with hypertension who have high self-efficacy exhibit greater willingness and ability to actively acquire knowledge about hypertension-related diseases, and their ego depletion levels are relatively lower. In light of this, healthcare professionals should prioritize older adults with hypertension who have low self-efficacy. Targeted interventions such as peer support programs, optimized goal-task alignment, and guidance to perceive positive benefits in disease management can be implemented to enhance these patients’ confidence in managing hypertension and stimulate their intrinsic motivation for self-regulation, thereby preventing or delaying the onset and progression of self-sustaining deterioration.

## 5 Conclusion

Older adults with hypertension exhibit high levels of ego depletion with heterogeneity. Through the latent class analysis, the ego depletion can be categorized into three groups: low depletion group, medium depletion group, and high depletion group. Key correlates include primary caregivers, hypertension classification, disease duration, and self-efficacy. Variations were also observed in total self-management intention scores and dimension-specific scores across different latent classes of older adults with hypertension. This suggests that healthcare professionals should prioritize associations and implement early targeted interventions to reduce patients’ ego depletion levels, thereby improving their overall quality of life. This study employed a cross-sectional design, which reveals the associations between variables but does not allow inference of causal direction or temporal sequence of the relationships. Meanwhile, this study used a convenience sampling method and recruited hospitalized older adults only from a single tertiary hospital. This population has systematic differences from community-dwelling older adults in terms of health status, social support, and other aspects, which may lead to the overestimation of the measured psychological burden and levels of self-management behaviors, thereby limiting the generalizability of the study results. Future research should include community-dwelling older adults and broaden the sample sources to improve the representativeness and generalizability of the findings.

## Data Availability

The raw data cannot be shared publicly to protect the confidentiality of the study participants, as approved by the Ethics Committee of Yijishan Hospital, Wannan Medical College. Requests to access the datasets should be directed to HT, 2954957052@qq.com.

## Author’s note

Special Project of the China Association of Gerontology and Geriatrics ‘Research Report on the Development of China’s Aging Work’ (CAGG2025ZX043).
